# Silver and Graphenic Carbon Nanostructures Differentially Influence the Morphology and Viability of Cardiac Progenitor Cells

**DOI:** 10.3390/ma13092159

**Published:** 2020-05-07

**Authors:** Anna Hotowy, Marta Grodzik, Marlena Zielińska-Górska, Natalia Chojnacka, Natalia Kurantowicz, Sławomir Dyjak, Barbara Strojny, Marta Kutwin, André Chwalibog, Ewa Sawosz

**Affiliations:** 1 Department of Nanobiotechnology and Experimental Ecology, Warsaw University of Life Sciences, 02-786 Warsaw, Poland; anna_hotowy@sggw.pl (A.H.); marta_grodzik@sggw.pl (M.G.); marlena_zielinska_gorska@sggw.pl (M.Z.-G.); n.chojnacka93@gmail.com (N.C.); natalia_kurantowicz@sggw.pl (N.K.); barbara_strojny@sggw.pl (B.S.); marta_kutwin@sggw.pl (M.K.); ewa_sawosz@sggw.pl (E.S.); 2 Institute of Chemistry, Military University of Technology, 00-908 Warsaw, Poland; slawomir.dyjak@wat.edu.pl; 3 Department of Veterinary and Animal Sciences, University of Copenhagen, 1870 Frederiksberg, Denmark

**Keywords:** cardiac progenitor cells, hierarchical nanoporous graphenic carbon, silver nanoparticles, chicken, nanobiotechnology

## Abstract

The characteristic features of nanomaterials provide rich opportunities for a broad range of applications due to their different physicochemical properties. Nanocolloidal silver and graphenic carbon materials differ in most physicochemical characteristics, except for their nanodimensions. Since there is a growing demand for stem cell therapies for coronary disorders, examining cardiac progenitor cells (CPC) in terms of their response to nanostructure treatment seems to be a reasonable approach. Morphological studies and viability assessments were performed with CPC in vitro, treated with small concentrations of silver nanoparticles (AgNP), hierarchical nanoporous graphenic carbon (HNC) and their mixtures. A viability test confirmed the morphological assessment of CPC treated with AgNP and HNC; moreover, the action of both nanomaterials was time-dependent and dose-dependent. For AgNP, between the two of the applied concentrations lies a border between their potential beneficial effect and toxicity. For HNC, at a lower concentration, strong stimulation of cell viability was noted, whereas a higher dosage activated their differentiation. It is necessary to perform further research examining the mechanisms of the action of AgNP and especially of unexplored HNC, and their mixtures, on CPC and other cells.

## 1. Introduction

The characteristic features of nanomaterials, namely small size and high specific surface area per mass unit, provide abundant opportunities for their use in biomedical applications [1]. Nanoparticles are used as therapeutic agents, antimicrobials, transfection vectors and fluorescent labels [2]; the broad range of applications is due to the extremely different physicochemical properties they can possess. As an excellent example, nanocolloidal silver nanoparticles (AgNP) and graphenic carbon materials differ in most of their physicochemical characteristics, except for their nanodimensions. Both have been extensively researched for possible therapeutic activity [3,4,5].

AgNP belongs to the longest studied and best-characterised nanoparticles. The first experiments concerned the antimicrobial properties of AgNP, and proved their antibacterial [6,7], antifungal [7,8] and antiviral [9,10] properties. The next showed their lower toxicity to eukaryotic cells in vitro [11,12,13] and to higher model organisms, namely nematodes and rats in vivo [14,15] compared to silver ions. For mouse [12] and human cell lines, silver nanoparticles at high concentrations (above 5 μg/mL) are shown to exert a cytotoxic effect. Interestingly, a low, nontoxic AgNP concentration (2.5 μg/mL and lower) for a short period (24 h) in the same experiments induced cell activation [11,12,13]. For silver nanoparticles obtained during “green synthesis” for pharmaceutical applications, antimicrobial activity and biocompatibility, or even in some cases protective action against oxidative stress, were noted for eukaryotic cell lines such as keratinocytes and fibroblasts, among others [3]. Surprisingly, there is a lack of data showing how these nanoparticles in low concentrations act on cells during a long-time culture. The influence of a range of concentrations below the toxic dosage, which may cause unexpected metabolic or physiological changes, are very interesting because they could bring a solution for existing health disorders. In the case of medicines, the toxicity can be determined to find the dosage which is safe and effective at the same time, and which may have the required effect on a living organism. Regarding AgNP, its toxicity and mechanisms have been extensively studied [16], in contrast to their potential beneficial influence at low concentrations. Our earlier studies on chicken model revealed that AgNP, after four weeks of oral administration, upregulates the expression of proangiogenic factors FGF2 and VEGFA in the heart [17]. Angiogenic properties of AgNP are also confirmed in mice and in endothelial cell line SVEC4-10 in vitro model [18]. However, it is still not clear whether this influence on the level of the whole organism is direct, or if it is caused by stimuli sent from the other organs.

Graphene is one of the carbon allotropes. The unique property of graphene flakes is the ratio of thickness to surface size that distinguishes this material from all the others. Graphene has unique physicochemical properties; its key feature is the high speed of electrons, which has a broad biological effect on different types of cells and tissues [19]. Through adhesion and binding to cell receptors, the charge of graphene can interfere with cell membranes, thereby blocking the access to nutrients, and leading to the activation of apoptotic mechanisms in cancer cells [20]. It has been shown that both surface chemistry and size play key roles in controlling biodistribution, toxicity and excretion of graphene, and, therefore, different graphene materials exert different effects on organisms [21]. The impact of functionalised graphene nanomaterials on living organisms was, therefore, investigated.

There are studies on the effect of graphene in oxidised form combined with silver nanoparticles. These nanocomposites are a promising agent with a broad spectrum of activity against methicillin-resistant *Staphylococcus aureus* strains. They can be used as platforms for the development of new materials capable of limiting the spread of microorganisms on biomedical devices and hospital facilities. These nanocomposites show improved antibacterial activity compared to silver nanoparticles alone [22,23]. Hierarchical nanoporous graphenic carbon (HNC) is a special type of graphenic materials provided so far only for heterogeneous catalysis, chromatography and energy storage applications. A simple nonchemical method of production allows one to obtain a highly porous material with a vesicular spatial structure, highly porous and free of harmful chemicals [24]. This nanostructure, with its properties of graphenic materials, seems to be a promising candidate for a carrier of active compounds, with AgNP among others.

Since there is a growing demand for stem cell therapies for coronary disorders, the examination of cardiac progenitor cells (CPC) in terms of their response for nanomaterial treatment is desirable even in respect to simple cell morphology, as an indicator for the first visible signs of cellular response. Morphological stem cell colony analysis is proven to contribute in the screening of quality maintenance of regenerative medicine products [25,26,27]; hence, even weak microscopic magnifications would be sufficient to register morphological changes on a cellular or colony level.

The present experiment was performed to clarify the effect of colloidal AgNP, HNC and their mixture on CPC originating from an eight-day-old chicken embryo. The study will assess how low concentrations of these nanomaterials affect CPC morphology and viability in the long-term culture, to verify the possibility of their potential regenerative applications.

## 2. Materials and Methods

### 2.1. Materials

#### 2.1.1. The Culture of Cardiac Progenitor Cells (CPC) from Embryonic Chicken Heart

##### Chicken Embryos

The fertilised eggs (*Gallus gallus*) of Hubbard strain chickens were supplied by a commercial, local hatchery (Marylka, Poland). The eggs were kept for 4 d at 12 °C. Prior to incubation, the eggs were sterilised by UV radiation for 30 s (UVKOR). The eggs were placed in an incubator with a controlled temperature and humidity, and automatic changing of the slope of the shelves (ALMD1 N5, FEST, Gostyn, Poland). The eggs were incubated for 8 days under standard conditions (37 °C, 60% Rh). The day when the eggs were placed into the incubator was designated as embryonic Day 0 (E0).

From Day 8, the chicken embryo (E8) cardiac progenitor cells, giving rise to heart muscle tissue, were isolated as follows.

##### Isolation of Heart

At Day 8 of embryonic development, the eggshell was cut open with surgical scissors sterilised in 70% ethanol. The content of the egg was poured onto a sterile Petri dish. The embryo deprived of foetal membranes was transferred to another Petri dish containing sterile, buffered with phosphate physiological saline (PBS, Gibco, Plisley, UK) and decapitated. The chest and abdomen were cut open, and the heart was isolated.

##### Isolation of Cells from Embryonic Heart

Dissected tissues were transferred to previously prepared sterile 2 mL Eppendorf tubes, containing 0.5 mL heated sterile trypsin (37 °C) (Trypsin-EDTA, Gibco) and left for 5 min to facilitate the disintegration of the tissue into single cells. Then, mechanical homogenisation was carried out by squeezing the tissue through sterile needles of smaller and smaller sizes (the last one of 0.45 mm × 12 mm) using a sterile 2 mL syringe. After obtaining a homogeneous suspension of cells, 1.5 mL of fresh culture medium heated to 37 °C—containing DMEM, high glucose, GlutaMAX^TM^ (Gibco), supplemented with 10% heat-inactivated foetal bovine serum FBS (Gibco) and 1% antibiotics-antimycotics (Gibco), consisting of penicillin, streptomycin and amphotericin B—was added to tubes with 0.5 mL of cell suspension to neutralise trypsin. The cell suspension was distributed to two wells of a 6-well plate, 1 mL into each of the wells. Then, 1 mL of fresh medium was added to each well. The plate was placed in a cell culture incubator (INC 108, Memmert, Schwabach, Germany) at standard conditions (temperature 37 °C, CO_2_ concentration 5%) and incubated until about 80% of confluence.

##### Cultures and Their Stabilisation in Culture Flasks Prior to the Experiment

When the confluence of primary cultures reached 80%, the cells were transferred under the laminar chamber, safety class II (Airstream, ESCO, Hatboro, PA, USA) from 6-well plates to 25 mL flasks. After 4 passages, flasks of cardiac progenitor cell culture confluent as close as possible to 80%, were selected for the experiment with several concentrations of nanomaterials in a culture medium.

#### 2.1.2. Nanomaterials

##### Hydrocolloidal Ag Nanoparticles

Hydrocolloidal silver nanoparticles (Nanokoloid, Warszawa, Poland) were produced with a proprietary (Patent 3883399), nonexplosive, high voltage method, using a high purity metal (99.9999%) and demineralised water. Colloid contained silver nanoparticles at a concentration of 50 µg/mL. The size of the silver nanoparticles ranged from 2 nm to 35 nm.

##### Hierarchical Nanoporous Graphenic Carbon Nanostructures

HNC nanostructures were provided by the Institute of Chemistry of the Military University of Technology (Warsaw, Poland). They are formed of very thin layers of mononuclear carbon and consist of shells (hollow carbon nanocubes) that are uniform in size, graphenic in nature, with hierarchical nanoporosity and a specific surface area of up to 1000 m^2^/g. HNC nanostructures are a new material obtained through simple physicochemical synthesis. The technology of production and physicochemical features of HNC have been described in detail by Dyjak et al. [24].

##### Suspensions of Nanomaterials in the Culture Media

Suspensions of carbon nanostructures HNC at a concentration of 1000 mg/mL were prepared in (i) deionised water and (ii) aqueous colloid of AgNP at a concentration of 50 µg/mL. The resulting suspensions were sequentially diluted in appropriate liquid to working concentrations of 10 µg/mL for HNC and 10 + 50 µg/mL for the combined HNC + AgNP. Furthermore, the third working suspension was AgNP at a concentration of 50 µg/mL. Before each application, suspensions were subjected to 30 min of sonication (ULTRON, model 908). Media with nanomaterials were prepared on an ongoing basis, just before replacing the media in the tested cultures. Working suspensions were added to the culture media at 20% of the final volume (1:5 dilution). For lower concentrations of nanomaterials, the medium was further diluted five times with pure medium.

### 2.2. Methods

#### 2.2.1. Zeta Potential Measurement

Zeta potential is a measure of the effective electrical charge on the surface of the nanoparticle. When a nanoparticle has a surface charge, it is determined by the concentration of ions with opposite charge near to the surface of the nanoparticle. This layer of oppositely charged ions moves with the nanoparticle. The zeta potential is a measure of the difference in potential between the fluid in which the particle is dispersed and the fluid layer containing oppositely charged ions that are bound to the surface of the nanoparticles. Particles with negative zeta potential bind to a positively charged surface and vice versa [28].

Aqueous suspensions of applied nanomaterials and their suspensions in the culture medium at concentrations of the content in the culture media, were prepared by diluting the working solution in deionised water, and then sonication (ULTRON, model 908, Olsztyn, Poland) for 30 min at room temperature. Analysis of the preparations was performed using the device ZetaSizer (Nano ZS 90, Malvern Instruments, Malvern, UK) with Software 7.11. Each sample of aqueous suspension was stabilised by 120 s at 25 °C. Then, three 20-fold measurements were made for each sample.

#### 2.2.2. pH Measurement

pH measurement can be used to determine the acidity or alkalinity of water solution, which is also a culture medium. Measurements were performed by electrochemical method on Orion Star A111 benchtop pH metre standard kit with a general-purpose pH/ATC electrode in triplicate after calibration on three standards: pH 1.0; pH 7.01; pH 10.00. After each measurement, the electrode was rinsed with deionised water and dried with a paper towel.

#### 2.2.3. Viability Test

To examine cell viability after incubation with nanostructures, PrestoBlue test (Life Technologies, Carlsbad, CA, USA) was applied. PrestoBlue is a reagent that is reduced by living cells, giving a colourful reaction that makes spectrophotometric reading possible. The test outcome does not reflect directly the rate of cell proliferation or their number because the reduction of the reagent is possible also for not dividing cells, being the reason why the measurement of the viability of all living cells is possible no matter whether their vital processes are rapid or very slow and the phase of the cell cycle.

CPCs of the fourth passage were inoculated into a 96-well plate and incubated overnight at 37 °C. The next day, the control medium was removed from the wells and replaced by media with nanomaterials that were applied in the experiment, adding 90 µL of the medium and then 10 µL of the PrestoBlue reagent (Invitrogen, United States) to the wells in 6 repetitions for each treatment. The same media were poured into empty wells to obtain the readings of blank samples. The plate was incubated for 30 min in a cell culture incubator, under standard conditions, then the fluorescence was measured in individual wells on a microplate reader (Infinite M200 TECAN, Tecan i-Control 1.4 software, Zurich, Switzerland). As the nanostructures had their own fluorescence, the results for blank samples (media + nanostructures) were subtracted from the results of real samples (medium + nanostructures + cells). The results developed in this way were subjected to statistical analysis. Fluorescence reading took place at a wavelength of 590 nm. The excitation wavelength was 560 nm. Fluorescence was read again after 2, 24 and 48 h of incubation.

#### 2.2.4. Giemsa and May-Grünwald Staining

From the flasks with cell culture, the medium was removed. Flasks were washed twice with non-sterile PBS. Approximately 1.5 mL of 4% paraformaldehyde (PFA, Sigma-Aldrich, Poznan, Poland) cooled to 4 °C, was poured into flasks which were allowed to stand in the refrigerator (4 °C) for 10 min. After this time, the flasks were again washed twice with non-sterile PBS. On the fixed cells, 1.5 mL of May-Grünwald dye was applied (Sigma-Aldrich). After 3 min, 1.5 mL of non-sterile PBS, was poured into flasks, mixed and left for an additional 5 min. Reagents were then removed, and the cells were rinsed with non-sterile PBS again. Then 1.5 mL of Giemsa dye was added (Sigma-Aldrich), diluted in phosphate buffer at a ratio of 1:9 and filtered. After 15 min, the dye was removed, and cells were washed three times with distilled water. Flasks were allowed to dry.

#### 2.2.5. Optical Microscopy

Pictures showing the morphology of cells in culture were taken using an inverted optical microscope (Olympus CKX41, Shinjuku, Tokyo, Japan) with the installed camera (ProgRes C12 plus, ProgRes Capture v. 2.8.0 software, Jenoptic, Jena, Germany).

#### 2.2.6. Transmission Electron Microscopy

Pictures showing the morphology and structure of nanomaterials were taken with a transmission electron microscope (TEM). Solutions of each kind of nanomaterial and the mixture of them were sonicated for 15 min at room temperature, and then droplets of samples were put on Formvar-coated 300 mesh Cu grids (Agar Scientific Ltd., Stansted, Essex, UK). The samples were dried at room temperature and observed using a JEM-2000EX TEM (JEOL, Akishima, Tokyo, Japan).

#### 2.2.7. Statistical Analysis

The data obtained from fluorescence measurements were analysed using one way and multi-way analysis of variance ANOVA, using Statgraphics Plus 4.1 (StatPoint Technologies, Warrenton, VA, USA). One-way ANOVA was applied to determine the influence of nanostructures on cell viability. Multivariate ANOVA served to additionally evaluate the influence of these materials during the time. To determine whether the differences were statistically significant, Fisher’s exact test was used. The results were presented as mean values. Differences between groups at *p* ≤ 0.05 were considered significant.

## 3. Results

### 3.1. Morphology and Structure of Nanomaterials

**Figure 1:**
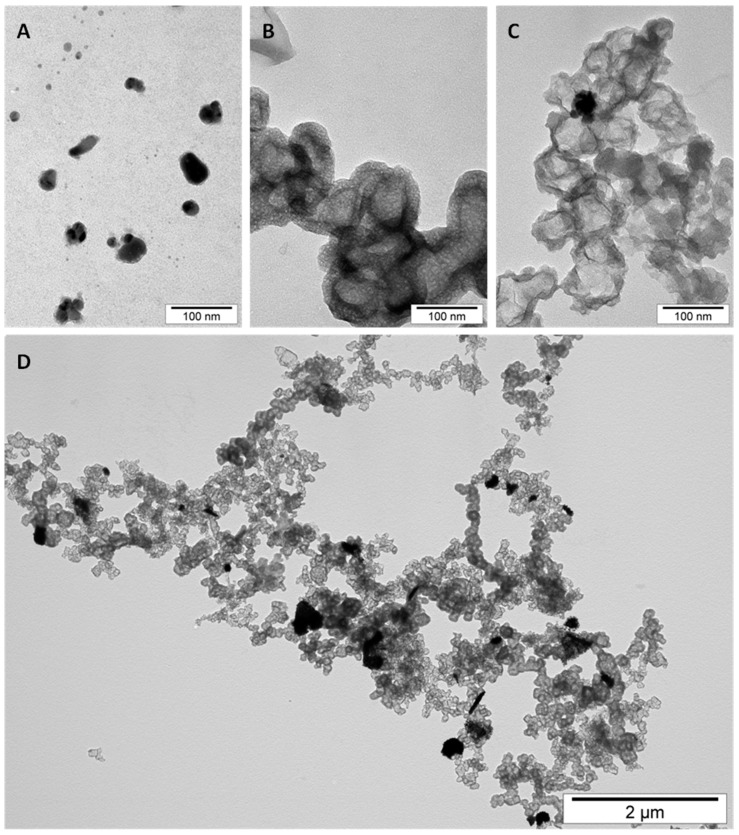
Morphology and structure of nanomaterials and their mixture: (**A**) silver nanoparticles; (**B**) carbon nanostructures (hierarchical nanoporous graphenic carbon (HNC)); (**C**,**D**) the mixture of silver nanoparticles and carbon nanostructure.

The TEM images of applied nanostructures and their mixtures are presented in Figure 1. It was seen that both nanomaterials substantially differed in shape and structure (Figure 1A,B). Moreover, these materials mutually influenced the morphology and agglomeration of each other (Figure 1C,D). Individual HNC shells were less compacted and seemed to be thinner and less porous.

In general, HNC seemed to be less spongy when mixed with AgNP colloid (Figure 1C). AgNP, in turn, agglomerated on the surface of HNC creating aggregates of smaller nanoparticles around the bigger ones (Figure 1C,D). As shown in Figure 1D, none of AgNP agglomerates existed separately from HNC.

### 3.2. Zeta Potential

The results of the measurements of nanostructures zeta potential in water solutions are presented in Table 1.

**Figure 2:**
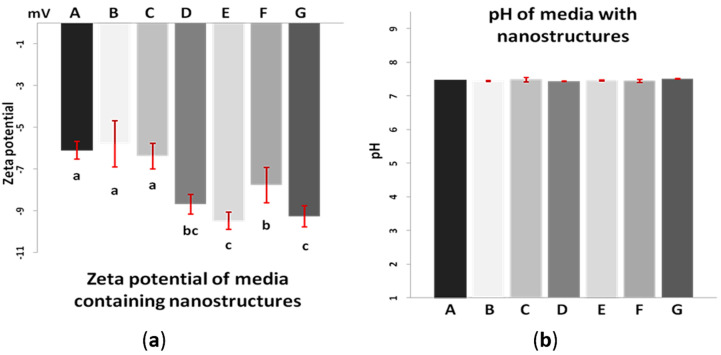
(**a**) Zeta potential of individual media with nanostructures. (**b**) pH values of individual media with nanostructures. (A) control medium; (B) medium with 2 µg/mL AgNP; (C) medium with 10 µg/mL AgNP; (D) medium with 0.4 µg/mL HNC; (E) medium with 2 µg/mL HNC; (F) medium with 2 µg/mL AgNP and 0.4 µg/mL HNC; (G) medium with 10 µg/mL AgNP and 2 µg/mL HNC. ^a–c^ Statistically significant differences (*p* < 0.0001).

Measured values of the zeta potential of the nanomaterials’ suspensions ranged from −11.2 to −20.0 mV. The results of the measurements of nanostructures zeta potential in culture media are presented in Figure 2.

### 3.3. pH of Culture Media

The results of pH measurements in individual culture media are presented in Figure 2b. They showed no significant differences.

### 3.4. Viability Test and Cells’ Morphology

The results of multifactor ANOVA showed that the time of nanostructure action on CPC is the main factor influencing their viability (Table 2). Regardless of the applied treatment, the most pronounced effects were observed between particular time points of CPC culture. The influence of time was so strong that in this type of statistical analysis, the effects of nanotreatment were not significant.

**Figure 3:**
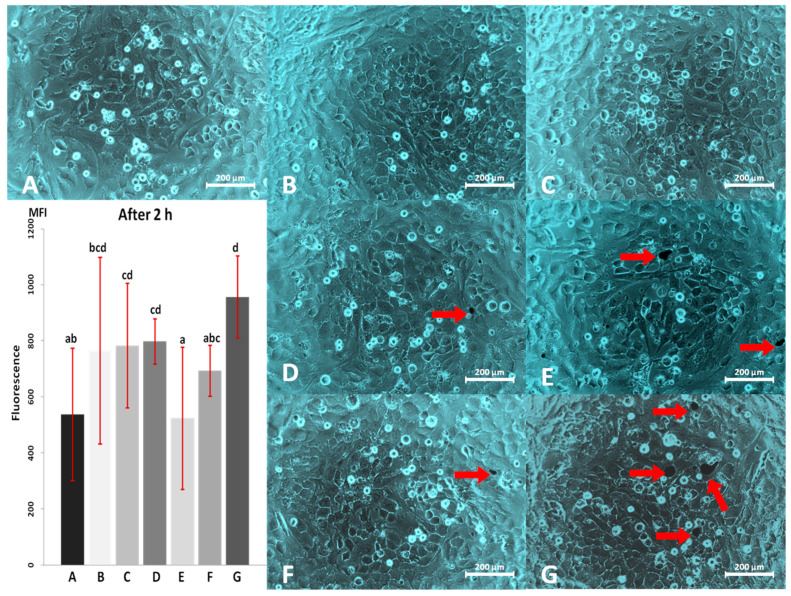
Cardiac progenitor cell morphology and viability after 2 h of culture with AgNP and HNC; (**A**) control culture; (**B**) culture with 2 µg/mL AgNP; (**C**) culture with 10 µg/mL AgNP; (**D**) culture with 0.4 µg/mL HNC; (**E**) culture with 2 µg/mL HNC; (**F**) culture with 2 µg/mL AgNP and 0.4 µg/mL HNC; (**G**) culture with 10 µg/mL AgNP and 2 µg/mL HNC. (**A**–**G**) Series of viability in individual experimental groups measured as fluorescence. ^a–d^ Statistically significant differences (*p* ≤ 0.05). Arrows indicate agglomerates of HNC. Magnification 100×. Scale bars 200 µm. MFI—mean fluorescence intensity.

Results of viability test and cells morphology after 2 h culture are shown in Figure 3.

CPC cultured in media containing the addition of AgNP at a concentration of 10 µg/mL (Figure 3C), the addition of HNC in the concentration of 0.4 µg/mL (Figure 3D) and the mixture of AgNP + HNC in a concentration of 10 + 2 µg/mL (Figure 3G) showed a higher level of viability than the cells in the control culture. Moreover, none of the treated groups after 2 h had lower viability than the control group. Regarding cells’ morphology on this stage, differences between experimental groups were not very strong and easily visible yet. Cultures having higher viability have also visibly the highest confluence.

**Figure 4:**
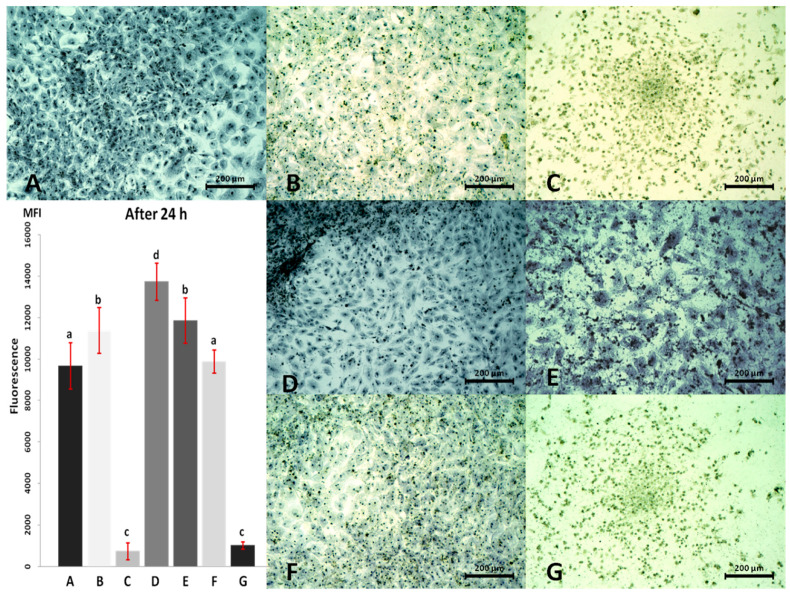
Cardiac progenitor cell morphology and viability after 24 h of culture with AgNP and HNC; (**A**) control culture; (**B**) culture with 2 µg/mL AgNP; (**C**) culture with 10 µg/mL AgNP; (**D**) culture with 0.4 µg/mL HNC; (**E**) culture with 2 µg/mL HNC; (**F**) culture with 2 µg/mL AgNP and 0.4 µg/mL HNC; (**G**) culture with 10 µg/mL AgNP and 2 µg/mL HNC. (**A**–**G**) Series of viability in individual experimental groups measured as fluorescence. ^a–d^ Statistically significant differences (*p* ≤ 0.05). Magnification 100×. Scale bars 200 µm. MFI—mean fluorescence intensity.

Results of the viability test and cell morphology changes after 24 h are presented in Figure 4.

Almost all of the CPC cultured in media containing an addition of any type and concentration of nanomaterials showed stronger differences in viability than the control cells, except the group receiving the mixture of AgNP + HNC in concentration 2 + 0.4 µg/mL (Figure 4F). Among the remaining groups, a higher viability level was seen in cultures receiving the addition of the AgNP (2 µg/mL; Figure 4B) and HNC (0.4 µg/mL and 2 µg/mL; Figure 4D,E). At the same time, both groups receiving AgNP in a high concentration (10 µg/mL; Figure 4C) alone or in mixture with HNC (Figure 4G) showed lower viability.

Differences in the cells’ morphology after 24 h were more pronounced than after 2 h culture. CPC that received lower concentrations of nanomaterials (alone and in a mixture) were slightly smaller but more numerous than the control cells (Figure 4B,D,F); in some places, they grew in a few layers (Figure 4D).

**Figure 5:**
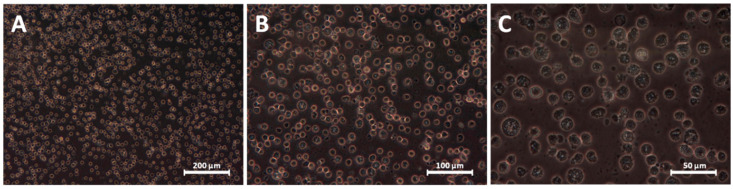
Morphology of cardiac progenitor cells cultured with the addition of 10 μg/mL AgNP after 24 h. Magnifications: (**A**) 100×; (**B**) 200×; (**C**) 400×.

Higher concentrations of nanomaterials caused even stronger changes in the cells’ morphology. CPC cultured in the presence of AgNP in a high concentration (10 µg/mL), alone and in a mixture, were much smaller, spherical and possessed puckered and pleated cell membrane (Figure 4C,G). In the picture after Giemsa-May-Grünwald staining they did not appear to be alive, but the picture of a living culture (Figure 5) showed they were still intact, and some of them conducted slowly vital processes. Cells treated with the high concentration of HNC alone (2 µg/mL) were, in contrast, much bigger, less confluent (Figure 4E) and some of them had a number of nuclei.

**Figure 6:**
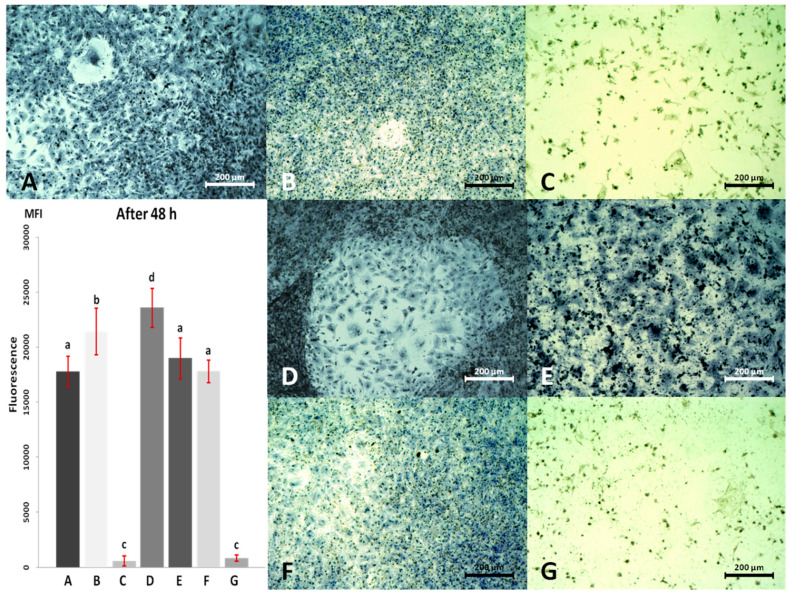
Cardiac progenitor cell morphology and viability after 48 h of culture with AgNP and HNC; (**A**) control culture; (**B**) culture with 2 µg/mL AgNP; (**C**) culture with 10 µg/mL AgNP; (**D**) culture with 0.4 µg/mL HNC; (**E**) culture with 2 µg/mL HNC; (**F**) culture with 2 µg/mL AgNP and 0.4 µg/mL HNC; (**G**) culture with 10 µg/mL AgNP and 2 µg/mL HNC. (**A**–**G**) Series of viability in individual experimental groups measured as fluorescence. ^a–d^ Statistically significant differences (*p* ≤ 0.05). Magnification 100×. Scale bars 200 µm. MFI—mean fluorescence intensity.

Results of the viability test and cells morphology after 48 h are presented in Figure 6.

After 48 h the deepening differences in viability emerged, compared to the control group: this meant that CPC receiving low concentrations of individual nanomaterials, i.e., 2 μg/mL of AgNP and 0.4 μg/mL of HNC, had a higher viability level (Figure 6B,D), while cell cultures receiving high concentration of AgNP (10μg/mL; Figure 6C,G) had a lower viability level (both alone and in mixture with HNC). In the group receiving the mixture of AgNP + HNC in a concentration of 2 + 0.4 µg/mL (Figure 6F) and HNC in high concentration (2 μg/mL; Figure 6E) no statistical differences in viability level were noticed.

Differences in the cells’ morphology after 48 h were even more pronounced than after 24 h culture. CPC that received lower concentrations of AgNP (alone and in the mixture; Figure 6B,F) were visibly smaller and denser than control cells and cells after 24 h cell culture (Figure 4B,F). Cells that received a low concentration of HNC (Figure 6D) looked similar to the control CPC for their dimensions and density, but compared with the 24 h culture (Figure 4D) they were smaller and more numerous.

**Figure 7:**
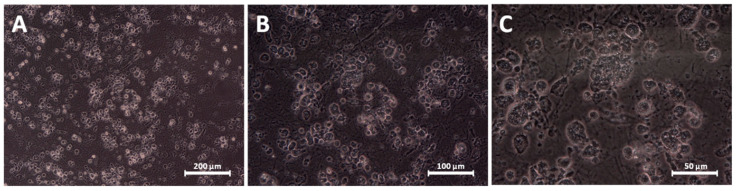
Morphology of cardiac progenitor cells cultured with the addition of 10 μg/mL AgNP after 48 h. Magnifications: (**A**) 100×; (**B**) 200×; (**C**) 400×.

CPC cultured in a high concentration of AgNP (10 µg/mL), alone and in the mixture, after 48 h were mostly dead. In the picture after Giemsa-May-Grünwald staining (Figure 6C,G), as well as in the picture of living culture (Figure 7), only irregular debris of cells and cell membranes can be seen. Probably if there were any living cells, they were singular. Cells treated with the high concentration of HNC alone (2 µg/mL; Figure 6G) remained much bigger, and their confluence was visibly higher than after 24 h (Figure 4G).

The results of recalculation of the viability rate of each experimental group on the relative viability fold change, compared to the control group, are presented in Table 3.

It can be seen that nanomaterials changed CPC viability. Although it lowered in time for each of the group as compared to the control, for some of them (low concentrations of separate nanomaterials), it was always from 1.2- to 1.5-folds higher than for the control culture. With regard to groups receiving the high concentration of AgNP alone or in mixture with HNC, even though relative viability of CPC after 24 h declined drastically (about 15 times), and again after the next 24 h fell approximately twice, after the first 2 h of culture, the highest values among all of the groups were seen.

## 4. Discussion

The characteristics of both nanomaterials (AgNP and HNC) differ substantially, which is the reason for the miscellaneous influence of these nanostructures on living cells and on each other. However, the interaction between them, clearly visible on TEM pictures (Figure 1C,D), was probably due mainly to the citric acid present in AgNP colloid. It is usually added in negligible concentration to all the colloidal solutions of metallic nanoparticles to prevent their agglomeration. Citric acid is the metabolite present in every living cell and taking part in the citric acid cycle (Krebs cycle) metabolising glucose and lactate, but its extremely low concentration (immeasurable) in the culture medium could not be harmful to cells as its addition was extremely low. Citric acid is commonly used as an additive and stabiliser of acidity in dietary products in much higher concentrations. There are several papers related to its application in human nutrition; for example, a paper by Pennistone et al. [29], where authors assessed citric acid amount in natural and commercially available juices and determined its range at the level of 1.4 g/oz, or an article by Chen et al. [30], where citric acid in a concentration of 10 g/L reduces decay and maintains the postharvest quality of fruits. Our results of pH measurements in media containing nanostructures, as can be seen in Figure 2b, showed no significant differences between individual media, no matter whether citric acid was present in added nanostructures’ solutions or not. Because the concentration of citric acid was negligible, and additionally was diluted by mixing of the colloid with the HNC solution, it came to be too low to prevent AgNP agglomeration. Probably, citric acid has also been adsorbed by the large surface of HNC, which became less compacted and slightly changed its structure. As in the culture media, there are a lot of different biological components that were probably able to act on nanomaterials in a similar way to citric acid in the colloid. However, this influence was not identical for HNC and AgNP. With regard to AgNP, the media components were sufficient to prevent aggregation, which confirms Figure 3C,G. CPC viability showed that cells reacted on the nanomaterial and its mixture merely the same way, that suggested a very similar active surface area of AgNP in both cases. Cellular responses to nanosilver were influenced by the physical and chemical nature of AgNP and varied depending on the cell type. Smaller AgNP (diameter = ~10 nm) penetrates into the cell, either by absorption into endosomes/lysosomes and endocytosis or by simple diffusion through the cell membrane. In contrast, larger-sized nanosilver or large nanosilver aggregates cannot enter the cell in this way, but can activate various signalling mechanisms through receptors that lead to oxidative stress [31,32].

In the case of AgNP applied in our experiment, the size of nanoparticles ranged between 3 and 50 nm, so their effect on CPC was probably multidimensional, and at low concentrations, the effect was not detrimental probably because of the sufficient mechanisms of exocytosis of the smaller nanoparticles, and detoxification of the harmful products of their action. Additionally, it has not been identified whether AgNP applied in mixture with HNC in the culture medium remained associated with graphenic nanostructure, to predict whether they could easily penetrate inside cells or not. It could not be observed by the mean of TEM because of the complex matrix of the mixture. For HNC, the situation was different; Figure 3D revealed bigger agglomerates of HNC than Figure 3F, where a slight amount of citric acid was added together with AgNP colloid.

Investigating studies on the activity of nanomaterials on living cells and organisms, they revealed the physical properties and the size of nanomaterials influenced the effect that could be observed [33,34,35]. Zeta potential measurement can help to verify whether particles in suspension are robust against aggregation. In our study, we performed such a measurement for water solutions and culture media containing nanostructures and their mixtures used in the experiment in particular concentrations. The absolute value of zeta potential higher than 30 mV testifies of the stability of a suspended substance in the solution. The results of measurements presented in Table 1 and Figure 2, indicate that the zeta potential of any of these suspensions did not exceed the absolute value of 30 mV. Moreover, measuring zeta potential in these conditions showed that nanostructures in culture media are even less robust against agglomeration than in water. It means that each of these nanomaterials separately and in mixture tend to form aggregates no matter whether they were suspended in water or in a culture medium. However, comparing suspensions in water with suspensions in the medium could be seen that the absolute value of zeta potential of the latter is lower. Furthermore, comparing these values for individual suspensions in culture media could be seen that the media containing HNC alone and in the mixture, no matter of concentrations, had significantly higher absolute zeta potential than media containing AgNP and pure medium. These values, however, were still too low to ascertain the stability of HNC nanostructures in the solution and prevent agglomeration. Such a process may lead to the loss of properties associated with the nano-dimensional nature of the nanoparticles, since aggregates of these materials have a reduced active surface area. To minimise the effect of aggregation, the suspension was additionally sonicated each time, just before the preparation of the media, however, for HNC this proved ineffective. Agglomerates of this nanostructure were clearly visible especially for its high concentrations after 24 and even more after 48 h of culture (Figure 4E and Figure 6E). Furthermore, in Figure 3E,G few agglomerates could be seen which had dimensions higher than nano. Regarding AgNP, Greulich et al. noted that they are unstable in terms of agglomeration; they reported, however, that complexing AgNP with the foetal bovine serum present in the culture medium stabilised the nanoparticles against aggregation [36]. On the basis of microscopic observations (Figure 3D, Figure 4D and Figure 6D), we can conclude that HNC agglomerates in the culture medium and its stabilisation by the foetal bovine serum present in the culture medium was also not effective. Although the zeta potential plays a key role in defining the stability of suspensions, it does not specify the size distribution of nanomaterials and their aggregates in the solution. The measurement of particle size with the DLS method is also offered by ZetaSizer Nano ZS 90. Although it is possible for stable colloids it is not proper for our nanostructures. When we tried to measure particle size the measurement could not be finalised because nanostructures continuously aggregated and sedimented. Accordingly, the exact size of the aggregates formed in cultures is not known. However, HNC agglomerates were much bigger than those of AgNP alone, since the biggest of them can be distinguished on the microscope image even in 100× magnification.

The influence of various concentrations of AgNP, HNC and their mixtures, on the viability and morphology of CPC, was tested over time. The applied concentrations were selected on a base of literature data and on a base of our previous experiments performed on microorganisms, living cells and higher model organisms (chicken embryo and broiler chicken). We have chosen concentrations about 3 to 5 times lower than those depicted as toxic by other researchers. It was found that the effect depended on the time of culture, as well as on the type and the dose of nanomaterial. Multiway ANOVA depicted the time as the main factor influencing cell viability (Table 2), so the comparison of changes in CPC viability and morphology in the experimental groups differing by treatments was performed at different time points.

Regarding the effect of the mentioned nanomaterials on CPC after 2 h, microscopic observation and the viability test revealed that none of the nanomaterials, irrespective of the concentration applied during this period of time, was harmful to CPC. Moreover, some of them seemed to enhance cell viability and promote their proliferation. After 2 h of the experiment in all of the groups (not excluding the control), few floating spherical cells detached from the surface were visible (Figure 3), however, this is a normal phenomenon, as a small percentage of the elderly, or abnormal cells always die. After this time, CPC morphology in all of the groups remained unchanged.

After prolonged culture (24 and 48 h), the results revealed longer exposure of CPC on mentioned nanomaterials, the stronger it influenced the cells’ morphology and viability. AgNP at a lower concentration did not affect the cells, whereas higher concentrations (either used alone or in combination with HNC) led eventually to their death. In turn, HNC at the lower and higher concentrations affected cell morphology, especially their dimensions. However, when the mixture of nanomaterials was applied, regardless of the duration of culture, the effects on morphology level were not noted.

Comparing the results with other studies which applied the mentioned or very similar nanomaterials and conducted the experiment on different types of cells can be ambiguous because the morphology and function of various cell lines differ considerably.

Results of the concentration-dependent cytotoxicity of silver nanoparticles published by Qin et al., on stem cells isolated from urine, showed that clear cytotoxicity of AgNP was noted after 24 h of exposure, starting with a concentration of more than 4 μg/mL [37]. A similar result was obtained by Greulich et al. who examined mesenchymal stem cells; they demonstrated that the concentration-dependent cytotoxicity started with 3.5 μg/mL after 7 days of exposure to AgNP [34]. What is important is that the concentration for eukaryotic cells was still higher than for the minimum inhibitory concentration of silver nanoparticles for microorganisms, namely 0.7 ng/mL for *Saccharomyces cerevisiae*, 0.35 ng/mL for *Escherichia coli* and 3.5 ng/mL for *Staphylococcus aureus* [38].

The dose of a substance can be defined as its quantity that reaches the biological system. It is directly related to the exposure that is the concentration of the substance in the medium, multiplied by the duration of contact [39]. Our results depict that CPC exposed to 2 μg/mL of AgNP for 48 h have an even higher viability rate than cells in the control culture (Table 3), and probably could survive this concentration of silver nanoparticles in culture medium for a longer time. Additionally, the proven antimicrobial properties of AgNP [6,7,8,9,10] could allow the reduction or exclusion of antibiotics normally applied as an addition to the medium in the culture.

Interpretation and comparison of the results of studies on HNC are quite difficult because so far only two publications on the effect of this nanomaterial on the living cells have been published [40,41]. The first of these works showed that cytotoxicity of this material, tested on human glioblastoma cells and fibroblasts, and on chicken red blood cells, increased with concentration and started from 10 μg/mL. In the case of glioma cells, the authors found that their death was due to the activation of apoptosis through the mitochondrial pathway, probably caused by the enhanced production of reactive oxygen species. This phenomenon has been noticed for glioblastoma cells even at a concentration of 10 μg/mL HNC in the culture medium, and was also dose-dependent, whereas at this concentration it was not observed for healthy cells [40]. In red blood cells from the chicken embryo, in turn, a concentration of 10 μg/mL HNC applied in phosphate-buffered saline (PBS) solution turned out to be toxic evoking cell haemolysis and deformation [41]. This effect could be due to the lack of protective effect of culture medium compounds. The results corresponded with earlier reports dealing with other carbon nanomaterials such as diamond, graphite or graphene. Liao et al. demonstrated that the graphene flakes exhibit low toxicity to red blood cells and fibroblasts [42]. Talukdar et al., in turn, demonstrated that the concentrations of the graphene nanoparticles of less than 50 μg/mL are relatively safe for most cell types [43]. These findings, taking into account the physical properties of HNC as having a very high relative surface area, encourages their application as nanocarriers in future experiments. With the progress of the culture, more and more agglomerates of HNC were observed; it was, however, not possible to determine whether HNC entered the cells, or was deposited on their surfaces. The high specific surface area of HNC is likely to block or prevent particles entering inside the cell, however, similar presumptions applied to graphene. Graphene did not enter the cell it gathered around, suggesting the existence of a high affinity for the cells [20]. On the other hand, some authors have reported the possibility of endocytosis of carbon nanomaterials, for example, Wang et al. observed graphene oxide in human fibroblasts, which increased with the progress of culture [44].

Confluence, appearance and the number of CPC treated with the mixture of AgNP and HNC at the concentration (2 AgNP + 0.4 HNC) μg/mL (Figure 4D and Figure 6D), were at a similar level as the control culture. Their appearance was more like a cell culture treated with AgNP at a concentration of 2 μg/mL than cells exposed to HNC at a concentration of 0.4 μg/mL. Shrinking, and a loss of adhesiveness was not observed. There are some studies on the different ways of silver nanoparticles’ action on living cells. Qin et al. found that cells treated with AgNP concentration of 2 μg/mL had an increased expression of key genes of differentiation. AgNP-induced actin polymerisation increased the tension of the cytoskeleton, resulting in osteogenic differentiation of stem cells isolated from the urine [35].

After 24 and 48 h treatments of CPC with a mixture of AgNP and HNC, at a concentration of (10 AgNP + 2HNC) μg/mL (Figure 4G and Figure 6G), there was a significant reduction in the number of cells. Cells did not undergo staining (neither the nucleus nor the body of the cell) which suggests that the cells were dead; therefore, it can be assumed that AgNP was primarily responsible for the cytotoxic effect on the cells.

Tests of the cells’ viability revealed interesting relationships. CPC of our experiment treated with HNC nanostructures at 0.4 μg/mL (Table 3) had a higher viability level than the control cells, and this difference persisted over time and declined very slowly. These changes were not observed in the case of cells treated with a higher concentration of HNC. This time, the viability level of CPC remained the same, as in the case of control culture (Table 3).

A stronger influence was seen at the level of the cells’ morphology. Cells treated with 2 μg/mL HNC in the medium were distinctly bigger than the other cells in the experiment. It can, therefore, be concluded that HNC, even at such a low concentration, affects cells and influences their viability level and morphological changes. CPC from our experiment treated with HNC nanostructures at 0.4 μg/mL (Table 3), stood out from the other groups, as their viability was the highest. One of the reasons why the viability level in this group was increased could be the activation of treated cells on the first day of the incubation with HNC, to neutralise the “foreign” component of the medium. The most surprising result was the strong activation of cells treated with HNC at the concentration of 0.4 μg/mL. Perhaps, this concentration most effectively affected cells, which after prolonged exposure (as shown in Figure 6D) were the most numerous (or most active). Probably, HNC is not a nanomaterial that inflicts death to cells but only reduces their adhesion to the surface. In Figure 4D, the upper layer of the cells seems to be detached from the bottom, together with HNC agglomerates. The reason for this phenomenon is not clear, however, cells might respond this way by treating HNC as a biocompatible scaffold and attaching to the nanostructure more willingly than to the bottom of culture flask. Moreover, it may be due to the limitation of transport through the cell membrane as well.

A factor that contributes to the fact that the culture of CPC with (2 AgNP + 0.4HNC) μg/mL was similar to the control culture could be the mutual neutralising effect of AgNP and HNC at this concentration, after 24 and 48 h culture (Figure 4A,F and Figure 6A,F). However, as observed from the fluorescence measurement, protective action on cell viability was not manifested in the increased levels of nanomaterials in the mixture. This effect could be due to the rising tendency of HNC to aggregate in higher concentrations, that can also be seen in Figure 4E and Figure 6E. More agglomerated material exerted a less neutralising effect on AgNP and less influence on growing cells also because it had a smaller specific surface available for the interaction with them. In case of application of AgNP at a concentration of 10 μg/mL and the mixture of nanomaterials (10 AgNP + 2HNC) μg/mL, the viability measurement confirmed the changes on cell morphology and confluence observed during cell culture. The level of viability of cells exposed to these factors was very low. As assumed above, the impact of AgNP was more pronounced and mainly responsible for the obtained effects. The reason for such a significant decline in viability could be structural and functional damage to the mitochondria, which ultimately resulted in cell death. Cell death, however, is not the only cause of the decline of viability levels, as it can be also a consequence of a big slowdown or limitation of their vital functions [45].

Summarising, the viability test results showed that the time is the strongest factor influencing cells viability, no matter of treatment applied. Moreover, the viability test results coincide with microscopic observations of CPC treated with AgNP at concentrations of 2 µg/mL and 10 µg/mL alone, as well as in mixture with HNC. They revealed a dose-dependent manner of AgNP action, where, between the two applied concentrations lies a border between their probable beneficial effect and toxicity. Moreover, it was shown that in the mixture, the action of AgNP is stronger than HNC. The same test indicated also that the cells treated with HNC at a concentration of 0.4 µg/mL had higher viability than the control cells over time. As regards the case of HNC at a concentration of 2 µg/mL, cell viability was almost the same as of the control group, but after prolonged culture, they were morphologically different, and the number of cells was slightly lower.

These phenomena are due to the fact that cell viability/morphology could be changed after prolonged exposure to nanomaterials because they tended to deposit with time on the surface of cells, that leads to closer contact and stronger influence on CPC.

## 5. Conclusions

AgNP and HNC at low concentrations upregulated CPC viability in vitro. High concentrations of AgNP drastically downregulated CPC viability, even applied in mixture with HNC. High concentrations of HNC did not affect CPC viability but strongly influenced their morphology and abundance. It is necessary to perform further research examining the mechanisms of action of mentioned nanostructures on CPC and probably other cells of a pluripotent nature as well.

## Figures and Tables

**Table 1:** The results of measuring the zeta potential of suspensions: of silver nanoparticles (AgNP), carbon nanostructures (HNC) and the mixture of silver nanoparticles and carbon nanostructure (AgNP + HNC).

Nanostructures	Concentration (µg/mL)	Zeta Potential (mV)
AgNP	2	−14.3
AgNP	10	−20.0
HNC	0.4	−11.2
HNC	2	−12.2
AgNP + HNC	2 + 0.4	−15.4
AgNP + HNC	10 + 2	−13.3

**Table 2:** The mean viability level of cardiac progenitor cells (CPC) culture in timepoints. (A) control culture; (B) culture with 2 µg/mL AgNP; (C) culture with 10 µg/mL AgNP; (D) culture with 0.4 µg/mL HNC; (E) culture with 2 µg/mL HNC; (F) culture with 2 µg/mL AgNP and 0.4 µg/mL HNC; (G) culture with 10 µg/mL AgNP and 2 µg/mL HNC. ^a–c^ Statistically significant differences (*p* < 0.0001). All the data presented as mean fluorescence intensity (MFI).

Time	Treatment							Mean Value in Time Point	Standard Error
	A	B	C	D	E	F	G		
2 h	538	766	783	798	524	693	956	723 ^a^	139
24 h	9680	11,381	745	13,749	11,866	9880	1019	8251 ^b^	139
48 h	17,815	21,449	464	23,609	19,004	17,830	843	14,248 ^c^	139

**Table 3:** Cardiac progenitor cells relative viability fold change depending on time and treatment, compared to the control group; (A) control culture; (B) culture with 2 µg/mL AgNP; (C) culture with 10 µg/mL AgNP; (D) culture with 0.4 µg/mL HNC; (E) culture with 2 µg/mL HNC; (F) culture with 2 µg/mL AgNP and 0.4 µg/mL HNC; (G) culture with 10 µg/mL AgNP and 2 µg/mL HNC.

Time	Treatment						
	A	B	C	D	E	F	G
2 h	1	1.42	1.46	1.48	0.98	1.29	1.78
24 h	1	1.18	0.08	1.42	1.23	1.02	0.11
48 h	1	1.20	0.03	1.33	1.07	1.00	0.05
